# White matter tract signatures of the progressive aphasias

**DOI:** 10.1016/j.neurobiolaging.2012.12.002

**Published:** 2013-06

**Authors:** Colin J. Mahoney, Ian B. Malone, Gerard R. Ridgway, Aisling H. Buckley, Laura E. Downey, Hannah L. Golden, Natalie S. Ryan, Sebastien Ourselin, Jonathan M. Schott, Martin N. Rossor, Nick C. Fox, Jason D. Warren

**Affiliations:** a Dementia Research Centre, UCL Institute of Neurology, London, UK; b Wellcome Trust Centre for Neuroimaging, UCL Institute of Neurology, London, UK; c Centre for Medical Image Computing, University College London, London, UK

**Keywords:** Primary progressive aphasia, DTI, Networks, White matter

## Abstract

The primary progressive aphasias (PPA) are a heterogeneous group of language-led neurodegenerative diseases resulting from large-scale brain network degeneration. White matter (WM) pathways bind networks together, and might therefore hold information about PPA pathogenesis. Here we used diffusion tensor imaging and tract-based spatial statistics to compare WM tract changes between PPA syndromes and with respect to Alzheimer's disease and healthy controls in 33 patients with PPA (13 nonfluent/agrammatic PPA); 10 logopenic variant PPA; and 10 semantic variant PPA. Nonfluent/agrammatic PPA was associated with predominantly left-sided and anterior tract alterations including uncinate fasciculus (UF) and subcortical projections; semantic variant PPA with bilateral alterations in inferior longitudinal fasciculus and UF; and logopenic variant PPA with bilateral but predominantly left-sided alterations in inferior longitudinal fasciculus, UF, superior longitudinal fasciculus, and subcortical projections. Tract alterations were more extensive than gray matter alterations, and the extent of alteration across tracts and PPA syndromes varied between diffusivity metrics. These WM signatures of PPA syndromes illustrate the selective vulnerability of brain language networks in these diseases and might have some pathologic specificity.

## 1 Introduction

The primary progressive aphasias (PPA) or ‘language-led dementias’ are important on clinical and neurobiological grounds (Gorno-Tempini et al., 2011; Grossman, 2010; Rohrer et al., 2010c). Clinically, PPA is associated with the selective but relentless erosion of language functions; and neurobiologically PPA illustrates regional vulnerability of brain language systems to neurodegenerative pathologies that are collectively characterized by abnormal protein accumulation. The spectrum of PPA is clinically, anatomically, and pathologically heterogeneous (Grossman, 2010). The canonical PPA syndromes comprise semantic variant PPA (sv-PPA), characterized by impaired knowledge of the meaning of words and objects in association with focal predominantly left anterior temporal lobe atrophy; nonfluent/agrammatic variant PPA (nv-PPA), characterized by speech production failure with apraxia of speech and/or agrammatism, associated with predominantly left-sided peri-Sylvian atrophy; and logopenic variant PPA (lv-PPA), characterized by prolonged word-finding pauses and impaired phonological memory without agrammatism, associated with predominantly left-sided temporo-parietal atrophy (Gorno-Tempini et al., 2011; Rohrer et al., 2009, 2010b).

Despite the recent formulation of new consensus diagnostic criteria for PPA (Gorno-Tempini et al., 2011), substantial nosological difficulties remain (Knibb et al., 2006). These include the frequent occurrence of overlap syndromes, clinicoanatomical convergence between syndromic subtypes and pathological heterogeneity (Rogalski et al., 2011a; Rohrer et al., 2008, 2011). A recent wealth of genetic and neuropathological data has enabled certain clinical PPA phenotypes to be correlated with particular pathological substrates: for example, sv-PPA is predominantly associated with TAR DNA-binding protein 43 (TDP-43) type C pathology and lv-PPA with Alzheimer's disease (AD) pathology (Grossman, 2010; Rohrer et al., 2011; Whitwell and Josephs, 2011). However, despite these advances, predicting tissue pathology or indeed making an accurate clinical diagnosis remains challenging for the diseases in the PPA spectrum. This is compounded by a paucity of robust in vivo biomarkers to detect disease onset and track disease progression. These issues in turn present challenges for planning future trials of disease-modifying therapies in PPA: such trials are likely to target specific pathologies and to seek to initiate treatments early in the disease course to minimize cognitive decline.

The recent identification of changes in large-scale intrinsic connectivity networks associated with neurodegenerative disease syndromes (Greicius et al., 2004; Seeley et al., 2009) suggests a potentially powerful framework for understanding the regionally-specific but distributed effects of PPA diseases. White matter (WM) tracts bind cortical hubs within neural networks, and WM tract changes are therefore likely to hold important information about brain network disintegration in neurodegenerative pathologies. However, though substantial evidence has been amassed concerning regional cortical profiles of PPA (Gorno-Tempini et al., 2004; Josephs et al., 2006; Mummery et al., 2000; Pereira et al., 2009; Rohrer et al., 2009, 2010b), relatively little information is available concerning changes in WM tracts within brain language networks produced by neurodegenerative disease (Agosta et al., 2010; Whitwell et al., 2010; Zhang et al., 2009). Previous WM tract studies in PPA (Acosta-Cabronero et al., 2011; Agosta et al., 2010, 2011; Galantucci et al., 2011; Schwindt et al., 2011) have shown considerable anatomic and methodological variability. There remain few detailed comparisons between PPA subtypes and other neurodegenerative diseases, and between gray matter (GM) and WM changes in these diseases. Besides facilitating diagnosis and tracking of PPA (Larsson et al., 2004; Schmierer et al., 2007) identification of WM tract signatures of PPA syndromes might improve our understanding of the pathophysiology of network disintegration in these diseases and could yield important insights into the molecular organization of vulnerable language networks (Raj et al., 2012; Warren et al., 2012; Zhou et al., 2012).

Here we set out to identify profiles of white matter tract degeneration in each of the canonical clinical subtypes of PPA using diffusion tensor imaging (DTI) with several diffusivity metrics and an anatomically unrestricted tract-based statistical approach. We hypothesized that characteristic signatures of WM tract degeneration underpin each PPA subtype within the distributed language network. We further hypothesized that these signatures distinguish PPA syndromes from each other and from other neurodegenerative diseases, in line with a core role for specific network disintegration in the pathogenesis of PPA.

## 2 Methods

### 2.1 Subjects

Consecutive patients fulfilling current consensus criteria (Gorno-Tempini et al., 2011) for a diagnosis of PPA were recruited from the Specialist Cognitive Disorders Clinic at the National Hospital for Neurology and Neurosurgery. Subjects underwent a structured clinical and neurolinguistic assessment and structural magnetic resonance imaging (MRI) to exclude significant WM disease or other focal cerebral lesions. For purposes of syndrome definition, a general neuropsychological assessment using standardized tests was performed. Behavioral tests are described in more detail in the Supplementary data (see “Description of behavioural tests”). Demographic and neuropsychological data were analyzed statistically in STATA 10 (Statacorp) using Student *t* test and Wilcoxin rank-sum tests of significance. In addition, cerebrospinal fluid (CSF) data (if available) were analyzed to assess the extent to which particular syndromes were likely to have underlying AD (versus non-AD) pathology. Total CSF tau (a measure of neuronal loss, as a nonspecific accompaniment of neurodegeneration) and CSF amyloid-beta_1–42_ (Aß_1–42_; a measure of amyloid deposition specific for AD pathology) were measured (Innotest platforms, Innogenetics, Ghent, Belgium). Local reference ranges for tau and Aß_1–42_ were used to assess the likelihood of underlying AD versus non-AD pathologies; cases deemed to have probable underlying AD pathology had tau >307 pg/mL and Aß_1–42_ <325 pg/mL (cutoffs derived from local data with 85% sensitivity for AD).

Ethical approval for the study was obtained from the local institutional ethics committee and all subjects gave written informed consent to participate in accordance with the Declaration of Helsinki.

### 2.2 MRI acquisition

Brain MRI data were acquired for all subjects on a Siemens Trio 3T MRI scanner using a 32-channel phased array head-coil (Siemens, Erlangen, Germany). Two 64-direction DTI sequences were acquired with a single shot, spin-echo echo planar imaging sequence (field of view: 240 mm; matrix: 96 × 96; yielding an isotropic voxel size of 2.5 × 2.5 × 2.5 mm; 55 contiguous axial slices; repetition time: 6800 ms; echo time: 91 ms; b value: 1000 s/mm^2^), augmented with parallel imaging acceleration to reduce susceptibility artifact. Nine sequences without diffusion weighting were acquired (b = 0 s/mm^2^). A sagittal 3-D magnetization prepared rapid gradient echo T1 weighted volumetric MRI (echo time/repetition time/inversion time = 2.9/2200/900 ms, dimensions of 256 × 256 × 208, voxel size of 1.1 × 1.1 × 1.1 mm) and a coronal fluid-attenuated inversion recovery sequence were acquired. For all subjects, volumetric MRI, DTI, and fluid-attenuated inversion recovery sequences were assessed visually in all planes to ensure adequate coverage and to exclude artifacts, unexpected pathology, or significant motion.

### 2.3 Diffusion image analysis

Raw diffusion weighted images were affine-aligned to the first corresponding b0 image using a linear image registration tool (FLIRT v5.5) within the FMRIB Software Library (FSL v4.1.5) (Cook et al., 2006; Smith et al., 2004). DTI volumes were then combined for tensor fitting using CAMINO and the tensor eigenvalues (λ1, λ2, and λ3; where axial diffusivity, AX = λ1), radial diffusivity (RD = (λ2 + λ3)/2), trace diffusivity, (TR = λ1 + λ2 + λ3) and fractional anisotropy (FA) were extracted at each voxel. After tensor fitting, images were processed with the tract-based spatial statistics pipeline (TBSS v1.1) (Smith et al., 2006). A general linear model was created incorporating disease group membership as the factor of interest and nuisance covariates of age, sex, disease duration (as a measure of disease severity), and total intracranial volume, calculated by summing GM, WM, and CSF acquired from segmentation of the structural images (using SPM8, as described below). The same model was fitted separately to FA, RD, AX, and TR and each PPA subgroup was contrasted with the healthy and disease control groups and with each of the other PPA subgroups. Statistical analysis was implemented using the permutation-based (nonparametric) ‘randomize’ tool within FSL with 5000 permutations generated for each test. A significance threshold (*p* < 0.05) was applied after correction for multiple comparisons using family-wise error (FWE) correction with threshold-free cluster enhancement (TFCE) (Smith and Nichols, 2009). Significant results were projected onto a study-specific mean brain registered to standard (Montreal Neurological Institute; MNI) space. To provide accurate anatomic localization a series of tract-specific masks were applied to the significant whole brain results. Masks reflected the anatomic location of major white matter structures generated using a previously published probabilistic WM tract atlas (Mori et al., 2004). A probability threshold of >20% was deemed acceptable in defining the boundaries for each mask. In total 14 masks were generated which included right and left inferior longitudinal fasciculus (ILF), superior longitudinal fasciculus (SLF), uncinate fasciculus (UF), anterior thalamic radiation (ATR), cingulum bundle (CB), corticospinal tract (CST), corpus callosum (CC), and fornix. Using fslstats further information on significance level and number of significant voxels was obtained from the already thresholded significant (FWE-corrected) results within each anatomic mask.

To compare the extent of tract involvement within each disease group using different diffusivity metrics (FA, AX, RD, and TR), the number of voxels within each tract identified as significant at the prescribed threshold using that metric was divided by the total number of voxels within each atlas derived mask and expressed as a percentage. We chose to express tract involvement predominantly using this threshold-dependent, extent-based measure rather than (for example) a mean diffusivity value across the whole tract for 2 reasons: first, averaging diffusivity values over tract masks would be strongly influenced by partial volume effects (e.g., inclusion of CSF in the mask) and/or crossing fibers; and second, the extent of tract involvement gives an indication of the anatomic distribution of change that is not captured using a mean diffusivity value. By focusing on thresholded extent maps, we aimed to focus more precisely on disease effects.

### 2.4 Gray matter analysis

Profiles of GM atrophy were derived using voxel-based morphometry with the New Segment (Weiskopf et al., 2011) and DARTEL (Ashburner, 2007) toolboxes of SPM8 (www.fil.ion.ucl.ac.uk/spm) under Matlab 7.0 (Mathworks, Sherborn, MA, USA). Segmentation, normalization, modulation, and smoothing of GM images were performed using default parameter settings (8 mm smoothing). Final DARTEL transformations were combined with affine transformations to MNI space. Nonparametric permutation testing was used to examine differences in regional GM volume between groups and included age, sex, total intracranial volume and disease duration as nuisance covariates. Maps of GM atrophy were overlaid onto an MNI152 standard brain after FWE correction (*p* < 0.05) with TFCE.

## 3 Results

### 3.1 Subject characteristics

Thirty-three consecutive patients with PPA (mean age 65.2 ± 7.9 years; 24 female) were identified. Most patients were characterized as having nv-PPA (*n* = 13), lv-PPA (*n* = 10), or sv-PPA (*n* = 10) in line with current criteria (Gorno-Tempini et al., 2011). Twenty age and sex comparable healthy control subjects also participated (mean age 64.7 ± 5.5 years; 12 female). Twenty age and sex comparable patients with a diagnosis of AD were also included (mean age 62.8 ± 5.0 years; 11 female) to act as disease controls. Thirty of 33 subjects completed a detailed neuropsychological assessment; the remaining 3 patients were unable to comply with behavioral testing, or declined and their syndromes were defined based on bedside clinical assessment alone. In addition we identified an additional 2 patients with a cognitive syndrome compatible with Granulin-associated aphasia (mean age 61 ± 14.1 years; 1 female; disease duration 2.8 ± 1.8 years) (Rohrer et al., 2010a) and were subsequently confirmed to have a proganulin (GRN) mutation (both exon 2 c31fs). We did not include these subjects in this group study because of the fact that this group has clinically distinct features and are likely to have specific profiles of WM change on a molecular basis. Demographic and neuropsychological data for all subjects are summarized in Table 1. There were no significant differences in age or sex between disease and control groups. Neuropsychological profiles were in keeping with the clinical diagnosis in each group.

CSF data were available on 31 (56%) of all 53 affected individuals (comprising 7 nv-PPA, 9 lv-PPA, 5 sv-PPA, and 10 AD). Two of 7 nv-PPA cases (29%) had CSF consistent with underlying non-AD pathology and 8 of 9 lv-PPA cases (89%) had CSF consistent with AD pathology. All sv-PPA cases had CSF consistent with non-AD pathology. All clinically diagnosed AD cases had CSF consistent with AD pathology.

### 3.2 PPA groups versus healthy controls and AD disease-controls

**Supplementary Fig. 1:**
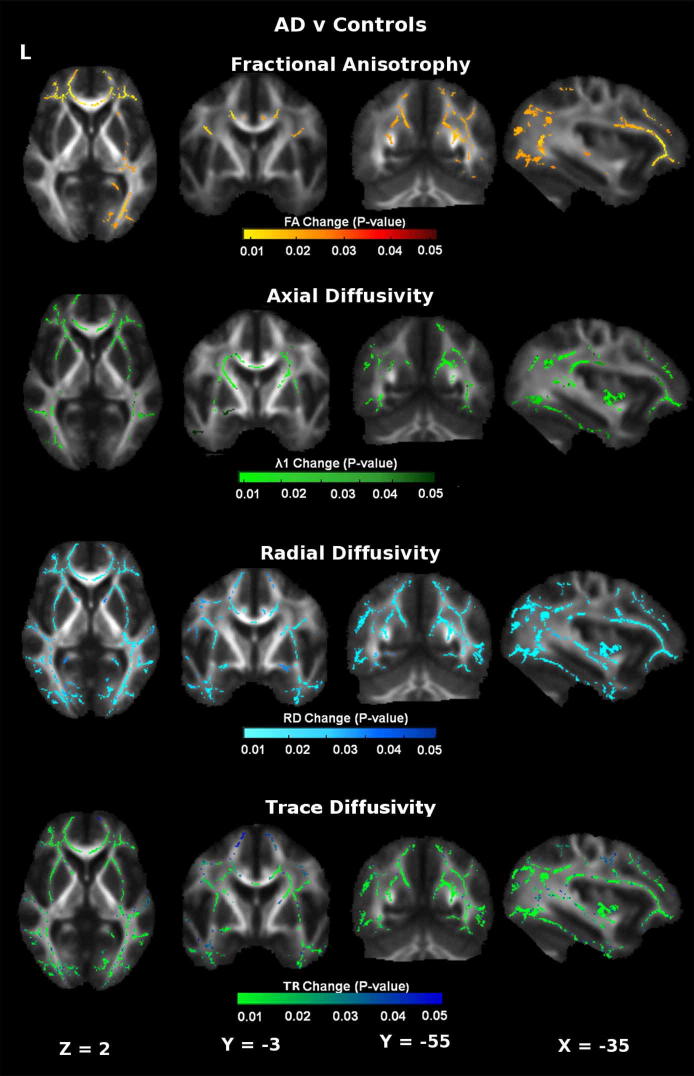
Patterns of altered diffusivity in Alzheimer's disease (AD) subjects versus healthy control group. Results are overlaid on representative sections (Montreal Neurological Institute coordinates shown below) derived from the average fractional anisotropy (FA) skeleton. For coronal and axial sections, the right hemisphere is shown on the left. The color bar (bottom) codes significance (family-wise error corrected *p* value). Abbreviation: RD, radial diffusivity.

**Fig. 1:**
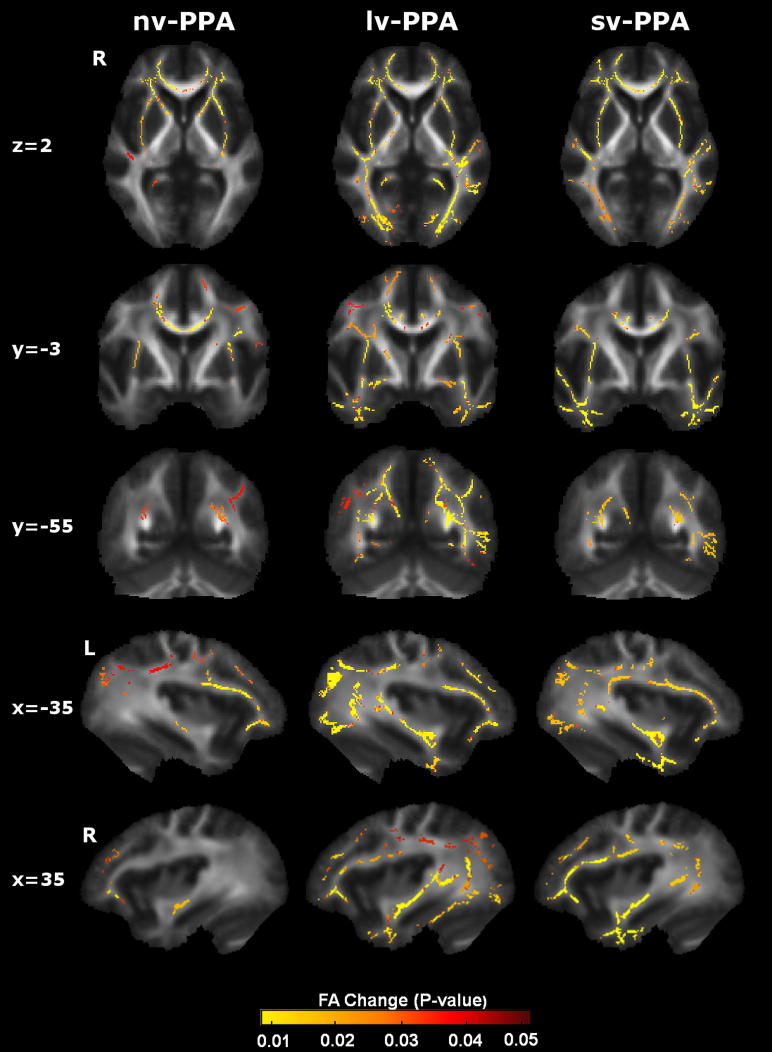
Patterns of decreased fractional anisotropy (FA) in primary progressive aphasia (PPA) groups versus healthy control group. Results are overlaid on representative sections (Montreal Neurological Institute coordinates shown on left) derived from the average FA skeleton. For coronal and axial sections, the right hemisphere is shown on the left. The color bar (bottom) codes significance (family-wise error corrected *p* value). Abbreviations: lv-PPA, logopenic variant PPA; nv-PPA, nonfluent/agrammatic variant PPA; sv-PPA, semantic variant PPA.

**Fig. 2:**
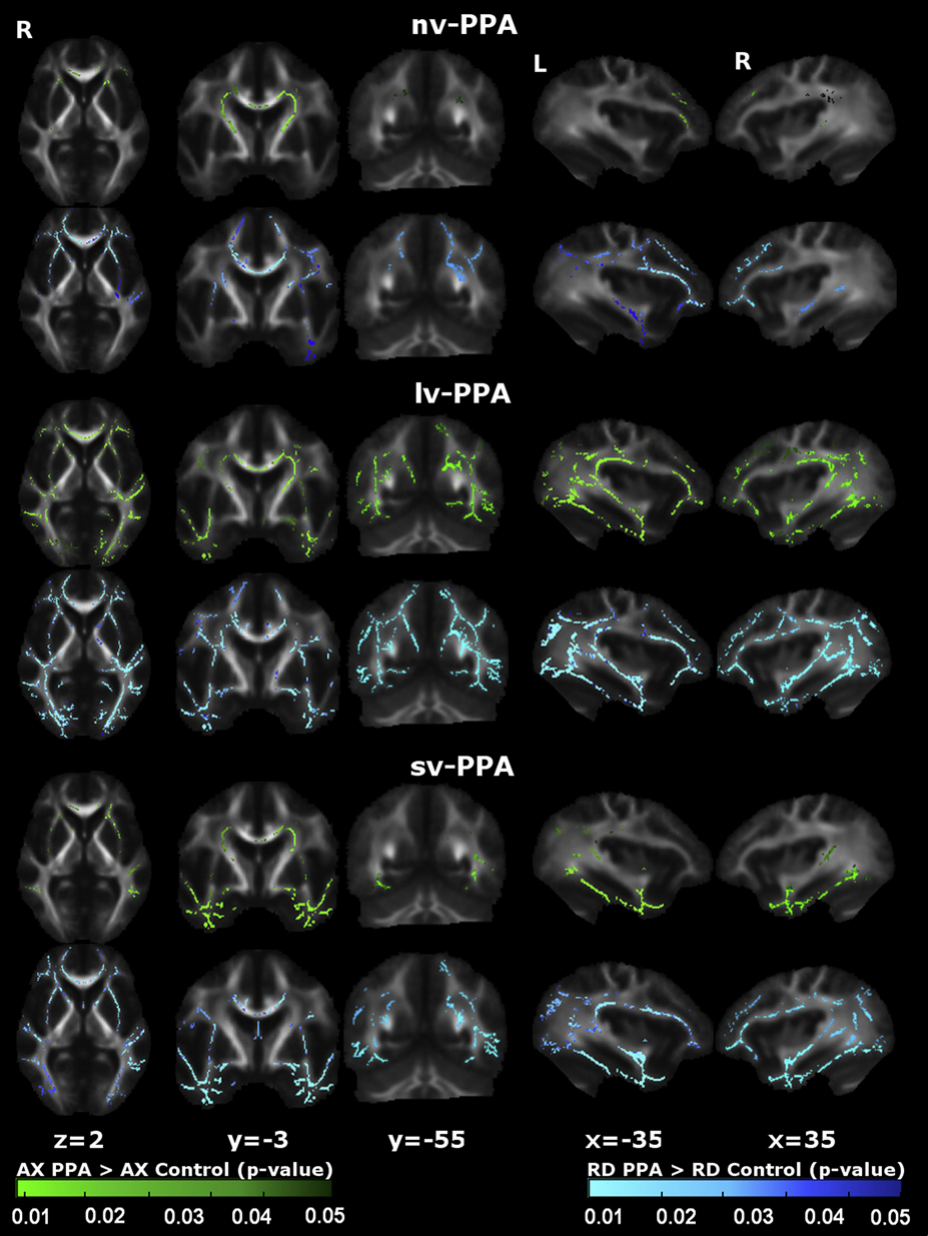
Patterns of increased axial and radial diffusivity in primary progressive aphasia (PPA) groups versus healthy control group. For each panel, the axial diffusivity (AX) map (green) is displayed on the top and the radial diffusivity (RD) map (blue) on the bottom. Results are overlaid on representative sections (Montreal Neurological Institute coordinates shown below) derived from the average fractional anisotropy skeleton. For coronal and axial sections, the right hemisphere is shown on the left. Color bars (bottom) code level of significance (family-wise error corrected *p* value). Abbreviations: lv-PPA, logopenic variant PPA; nv-PPA, nonfluent/agrammatic variant PPA; sv-PPA, semantic variant PPA.

**Fig. 3:**
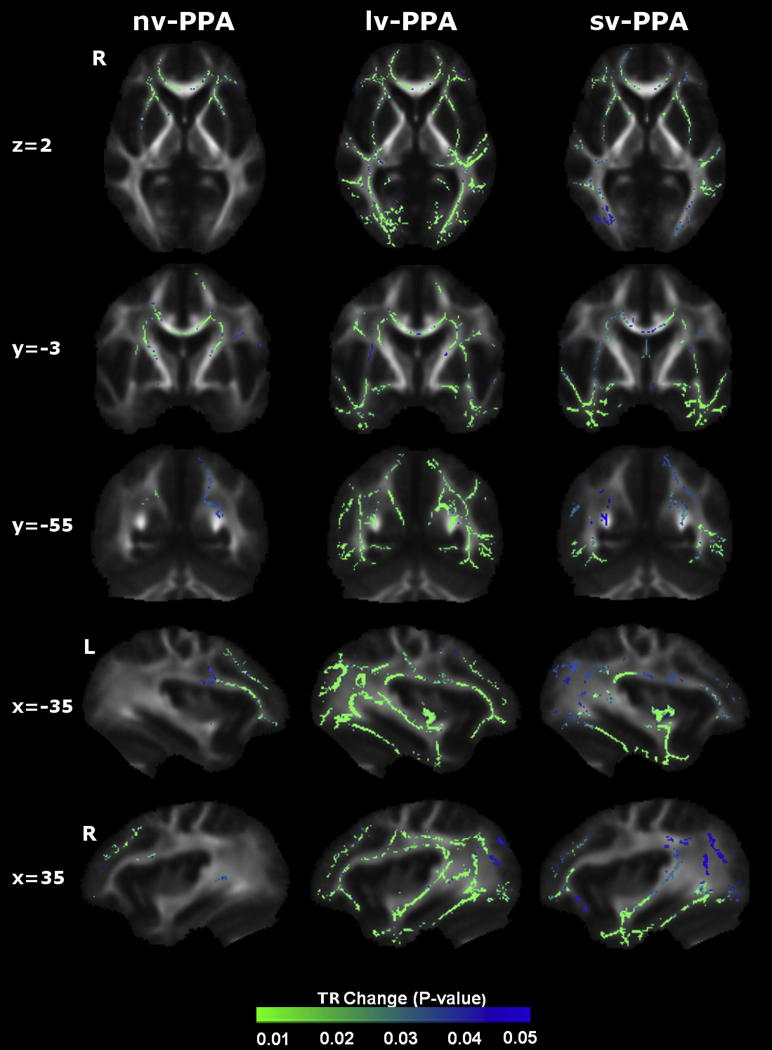
Patterns of increased trace diffusivity in primary progressive aphasia (PPA) groups versus healthy control group. Results are overlaid on representative sections (Montreal Neurological Institute coordinates shown on left) derived from the average fractional anisotropy skeleton. For coronal and axial sections, the right hemisphere is shown on the left. Color bars (bottom) code level of significance (family-wise error corrected *p* value). Abbreviations: lv-PPA, logopenic variant PPA; nv-PPA, nonfluent/agrammatic variant PPA; sv-PPA, semantic variant PPA; TR, trace diffusity.

**Fig. 4:**
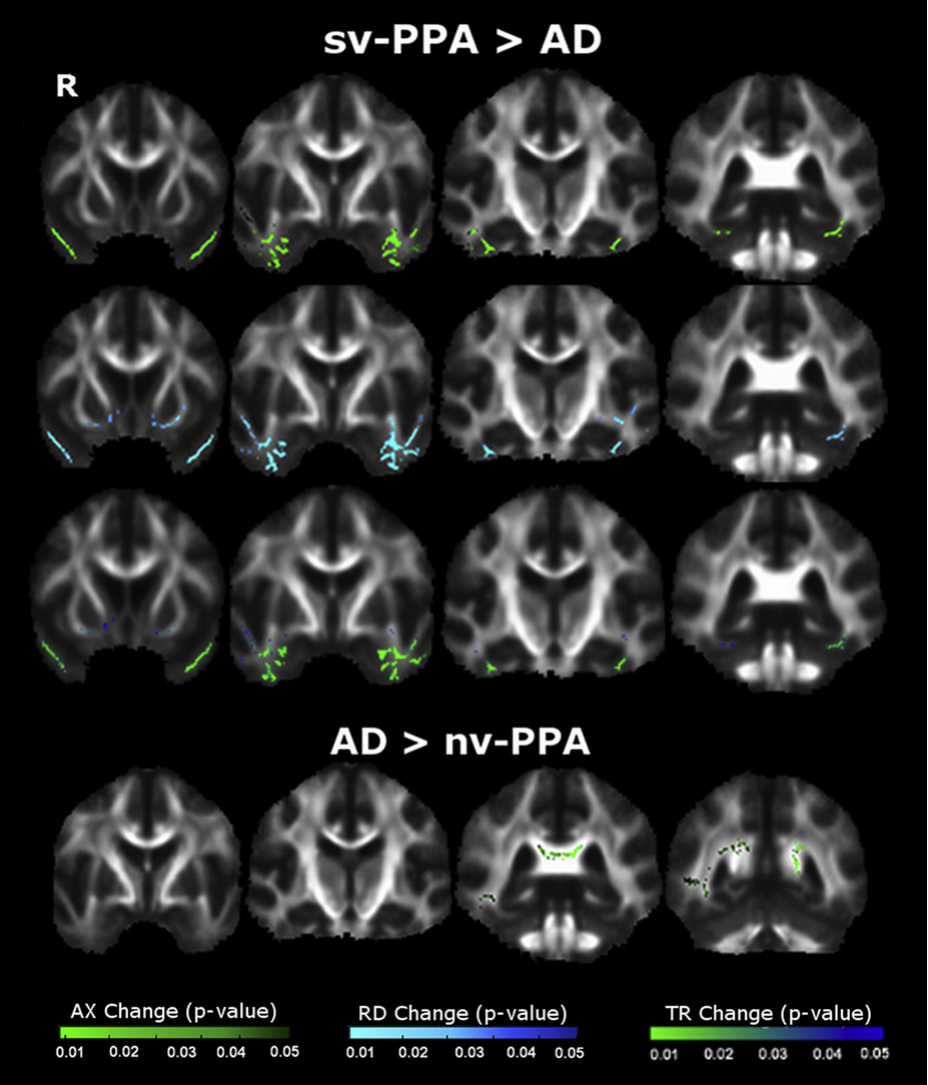
Comparison of white matter degeneration between primary progressive aphasia (PPA) groups and Alzheimer's disease (AD) group. Top panel displays patterns of increased axial diffusivity (AX) (top), radial diffusivity (RD) (middle), and trace diffusivity (TR) (bottom) in semantic variant PPA (sv-PPA) compared with AD. Bottom panel displays patterns of increased AX in AD compared with nonfluent/agrammatic variant PPA (nv-PPA). Results are overlaid on representative sections derived from the average fractional anisotropy skeleton. The right hemisphere is shown on the left. Color bars (bottom) code level of significance (family-wise error corrected *p* value).

WM tract alterations were identified for each PPA syndromic group compared with healthy control (Figs. 1, 2, and 3) and AD disease-control (Fig. 4) groups. Maps of altered diffusivity for AD disease-controls compared with healthy controls are presented in Supplementary Fig. 1. Individual tracts are listed and their involvement in each PPA syndrome relative to healthy controls is quantified in Table 2; quantitative data relative to the AD group are presented in Supplementary Table 1. Here, we first consider the overall profile of WM tract involvement in each syndrome, taking all diffusivity metrics into account (differences in the pattern of involvement across metrics are considered below).

Compared with healthy subjects, the nv-PPA group had WM tract degeneration in both cerebral hemispheres predominantly involving the left anterior frontal lobe (UF) and subcortical (ATR, CST) projections; in the comparison of nv-PPA with AD, the AD group showed more extensive posterior hemisphere WM tract degeneration (splenium CC, fornix, right ILF, right SLF). The profile of WM tract degeneration was most marked in anterior-dorsal left UF and anterior left ATR with less marked involvement of right hemispheric and posterior WM tracts. The greatest degree of WM tract change in nv-PPA (based on the total number of significant voxels across all tracts) was detected using RD (5.6% of tracts affected), followed by FA (5.1%), and AX (3.2%). Compared with healthy subjects, the lv-PPA group had extensive WM tract alterations within bilateral fronto-temporo-parietal regions, more marked posteriorly and within the left hemisphere with involvement of both dorsal and ventral WM tracts (ILF, UF, SLF, CB, ATR, fornix) and also CC; when compared with the AD group, no significant WM tract differences were observed. The profile of WM tract degeneration was most marked in left ILF, left CB, fornix, and left UF. The greatest degree of WM tract change in lv-PPA was detected using RD (10.6% of tracts affected), followed by FA (8.8%), and AX (8.8%). Compared with healthy subjects and AD patients, the sv-PPA group had WM tract degeneration involving both hemispheres with a particular emphasis on fronto-temporal (UF, ILF) WM tracts. The profile of WM tract degeneration was most significant within anterior-ventral fibers of bilateral UF and ILF with less significant involvement of dorsal and posterior WM tracts. The greatest degree of WM tract change in sv-PPA was detected using RD (8.5% of tracts affected), followed by FA (7.7%), and AX (3.8%).

### 3.3 Comparisons between PPA groups

**Fig. 5:**
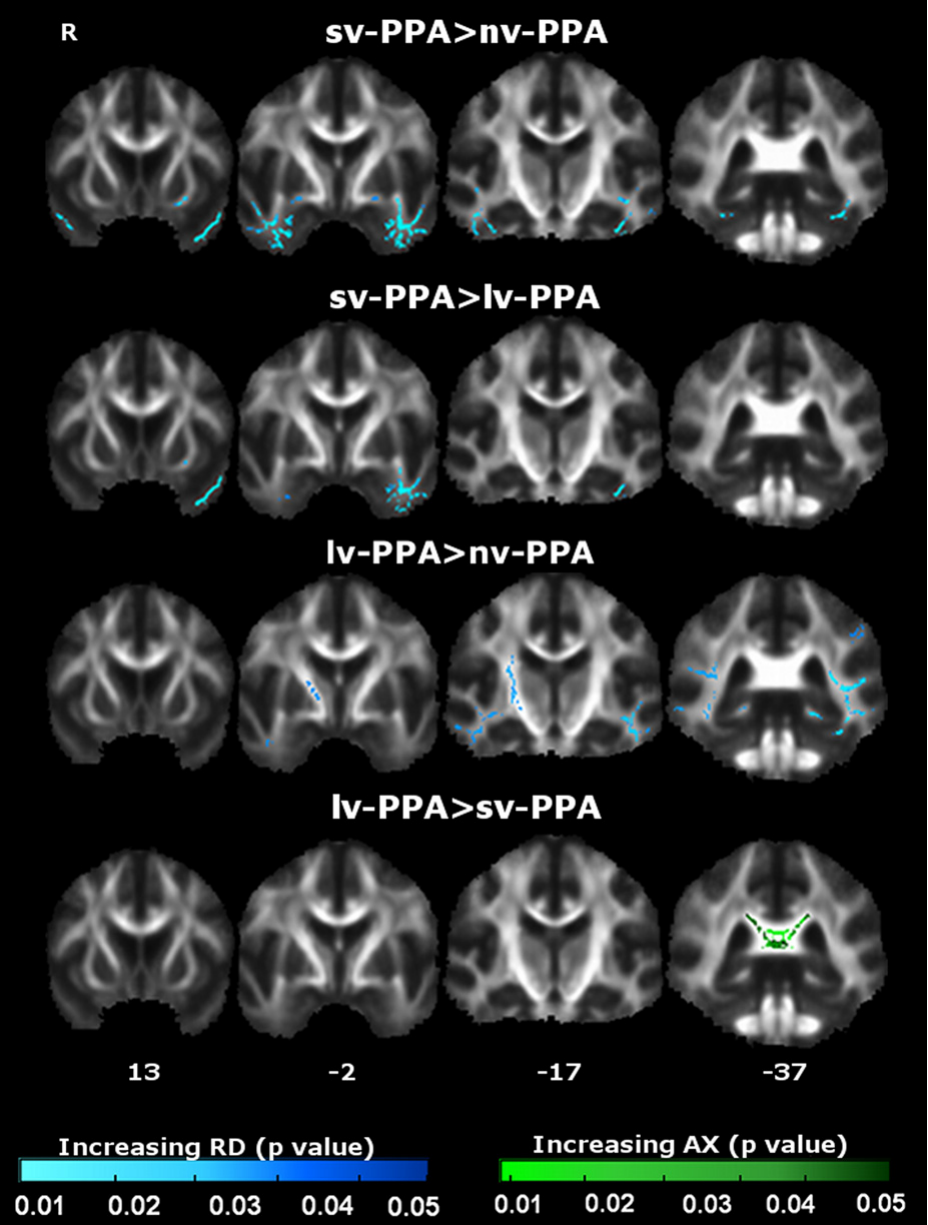
Comparison of white matter (WM) tract degeneration between primary progressive aphasia (PPA) syndromic groups. Each tile shows significant WM tract (family-wise error corrected *p* < 0.05) differences between groups as measured by increased axial diffusivity (AX) and radial diffusivity (RD). The right hemisphere is shown on the left. Montreal Neurological Institute coordinates are indicated below. Color bars (bottom) code level of significance (family-wise error corrected *p* value). Abbreviations: L, left; lv-PPA, logopenic variant PPA; nv-PPA, nonfluent/agrammatic variant PPA; sv-PPA, semantic variant PPA.

WM tract differences between PPA syndromic groups were also identified (Fig. 5); quantitative data are presented in Supplementary Table 2.

sv-PPA was associated with greater alterations in bilateral ILF and UF (left > right) relative to nv-PPA; and in bilateral ILF (left > right) and left UF relative to lv-PPA. lv-PPA was associated with more posterior bilateral WM tract alterations including ILF, SLF, UF, CC, CB, ATR, and CST relative to nv-PPA; and alterations in the splenium of CC relative to sv-PPA.

### 3.4 Individual subject DTI data

**Supplementary Fig. 2:**
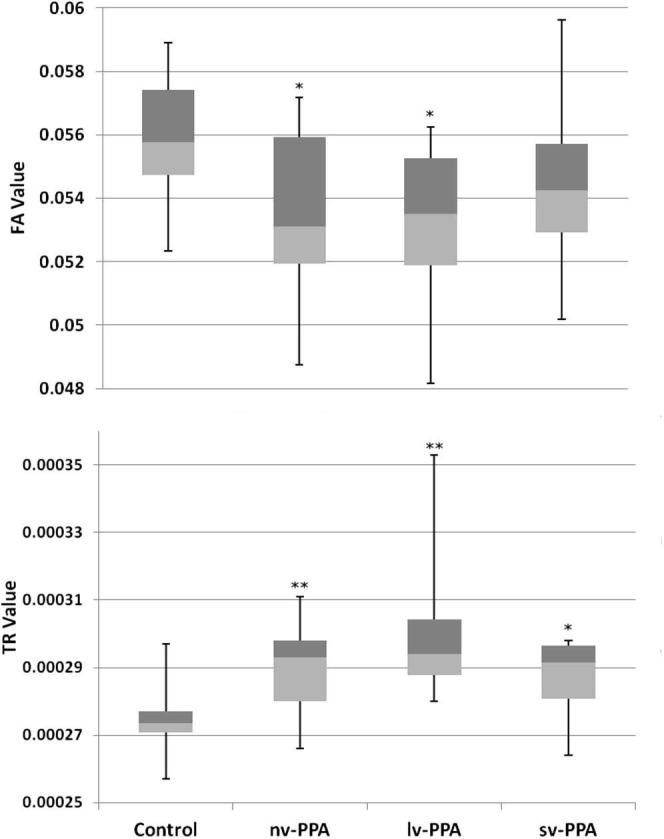
Box plots of individual subject mean fractional anisotropy (FA) and trace diffusivity (TR) values within superior longitudinal fasciculus. * = *p* < 0.01 versus healthy controls; ** = *p* < 0.001 versus healthy controls. Abbreviations: lv-PPA, logopenic variant PPA; nv-PPA, non-fluent/agrammatic PPA; PPA, primary progressive aphasia; sv-PPA, semantic variant PPA.

In light of the wide variation of group-level values between DTI metrics for SLF, a key language pathway (see Table 2), we also examined individual subject FA and TR data within SLF (results presented in Supplementary Fig. 2). The spread of individual values was somewhat wider for nv-PPA than for other PPA groups, though significantly different with respect to the healthy control group.

### 3.5 Gray matter analysis

**Fig. 6:**
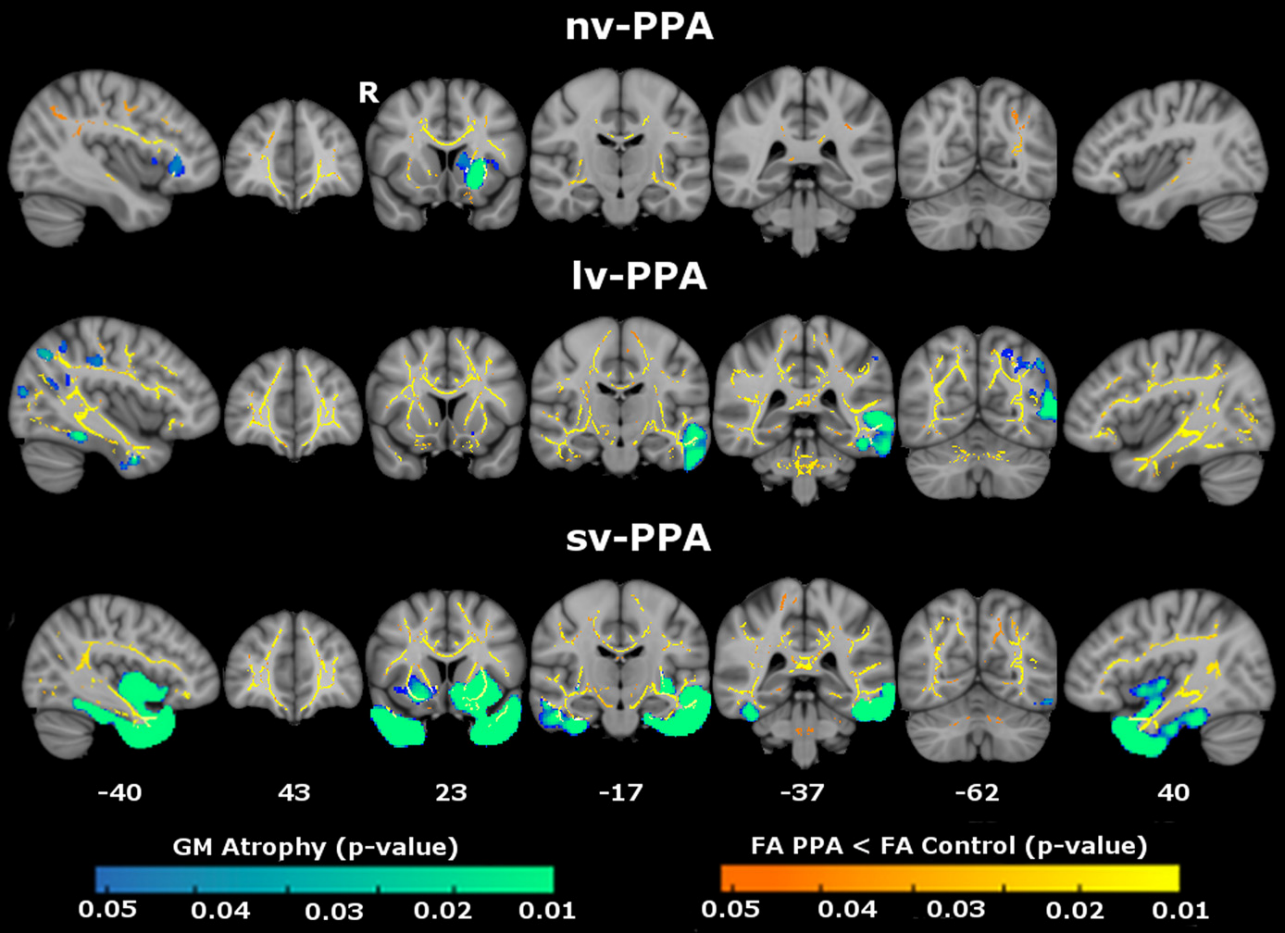
Maps of gray matter (GM) atrophy (blue-green) and reduced white matter fractional anisotropy (FA) (orange-yellow) in primary progressive aphasia (PPA) syndromic groups compared with healthy controls overlaid on a MNI152 template brain. GM and FA maps are thresholded at *p* < 0.05 after family-wise error correction with threshold-free cluster enhancement. Montreal Neurological Institute coordinates are displayed. Color bars (bottom) code family-wise error corrected *p* values (blue-green) for GM atrophy and FA change (red-yellow). Abbreviations: lv-PPA, logopenic variant PPA; nv-PPA, nonfluent/agrammatic variant PPA; R, right; sv-PPA, semantic variant PPA.

In the voxel-based morphometry analysis, each PPA syndromic group showed the anticipated profile of regional GM loss compared with the healthy control group (Fig. 6). sv-PPA was associated with bilateral but predominantly left-sided atrophy of antero-inferior temporal and orbitofrontal cortices. nv-PPA was associated with atrophy of left inferior frontal and opercular cortex. lv-PPA was associated with more extensive left hemispheric atrophy involving inferior and posterior-superior temporal lobe, temporo-parietal and temporo-occipital junctions. Regions of WM tract alteration closely neighbored atrophic cortical regions within each syndrome (i.e., left UF and left insular cortex in nv-PPA, left ILF and left temporo-parietal cortex in lv-PPA, anterior ILF and anterior temporal cortices in sv-PPA). However the tract alterations were spatially more distributed, extending beyond the zones of GM loss. This apparent disparity was most evident for lv-PPA, and less evident for nv-PPA and sv-PPA. The extent of WM tract changes varied with regard to the diffusivity metric used across all disease groups, with RD being associated with more extensive changes than AX or FA.

## 4 Discussion

We have identified signatures of WM tract degeneration across the PPA spectrum in relation to healthy controls and other neurodegenerative syndromes. WM tract differences also emerged on direct group-wise comparisons of PPA syndromes, suggesting relative specificity of profiles for particular syndromes and pathologies. Broad anatomical profiles of WM tract degeneration were identified for each of the disease groups here. The profiles of WM tract damage show some convergence with previous studies of WM tract involvement in PPA (Acosta-Cabronero et al., 2011; Agosta et al., 2010, 2011; Galantucci et al., 2011; Schwindt et al., 2011; Whitwell et al., 2010) (see Table 3); however, a number of additionally involved WM tracts were also identified for each of the syndromic groups in the present study, and there was substantial overlap between PPA syndromes. On visual inspection, WM damage appeared more widespread compared with areas of GM damage. These findings provide further evidence that WM tract metrics constitute markers of neural network disintegration across the PPA spectrum and might be helpful in tracking disease development.

The WM tract profiles delineated here help to define network-level substrates for the PPA syndromes. These profiles align with patterns of network breakdown previously identified in sv-PPA, nv-PPA, and lv-PPA (Seeley et al., 2009) and with language pathways proposed in the healthy brain (Saur et al., 2008). Here, nv-PPA was associated in particular with more marked involvement of anterior and subcortical tracts and relative sparing of more inferior temporal lobe tracts; this would be consistent with previous evidence concerning the neuroanatomic substrates of articulatory, phonemic, and grammar processing (Katanoda et al., 2001; Skosnik et al., 2002). Previous studies have demonstrated prominent involvement of left SLF in nv-PPA (Agosta et al., 2011; Galantucci et al., 2011; Schwindt et al., 2011); though involvement of left SLF was also demonstrated here, there was substantial variability in significance levels across DTI metrics (see Table 2) and a somewhat wider spread of individual subject FA data within the SLF in nv-PPA compared with other PPA groups (see Supplementary Fig. 2). The voxel-wise group data (Figs. 1, 2, and 3) also demonstrate involvement of the left SLF in the nv-PPA group, most prominently the anterior/frontal fibers; this is consistent with previous work showing that fiber tracts with a more anterior distribution (such as the arcuate fasciculus) are relatively more vulnerable in nv-PPA (Galantucci et al., 2011). This profile also provides a substrate for executive dysfunction in the present and previous nv-PPA cohorts (Rohrer et al., 2010b). sv-PPA here was associated in particular with involvement of ILF and UF: these tracts could support the putative anterior temporal–inferior frontal network mediating semantic and evaluative processing of words and nonverbal objects (Awad et al., 2007; Parker et al., 2005; Scott et al., 2000). lv-PPA was associated with involvement of both dorsally-directed (SLF, CB) and ventrally-directed (ILF) tracts, prominently including more posterior temporo-parietal projections and similar to the pattern seen in typical AD (Acosta-Cabronero et al., 2011); these widely distributed tracts are likely to support a distributed network that in the dominant hemisphere mediates phonological working memory and praxis and other parietal functions (Awad et al., 2007; Simon et al., 2002) and which has been previously implicated in the lv-PPA syndrome (Gorno-Tempini et al., 2011; Rohrer et al., 2010b). Though ILF involvement has been inconsistently reported in lv-PPA, identification of ILF in the lv-PPA group here is consistent with the known engagement of the ventral language network in lexical retrieval (Wong et al., 2011) and with previous studies of lv-PPA linking lexical retrieval deficits with more ventral GM atrophy (Wilson et al., 2010). Despite their distinct clinical phenotypes there was no significant difference in WM tract involvement when the lv-PPA and AD groups were directly compared; this suggests at least partial overlap of WM tract degeneration in these syndromes, in keeping with a common underlying disease pathology.

It is noteworthy that, for each of the PPA syndromes, WM tract alterations occurred in proximity to regions of GM atrophy but also extended more widely within each cerebral hemisphere (see Fig. 6). Though caution is needed in comparing different neuroimaging modalities and in calibrating for the effects of local atrophy on DTI metrics, it is unlikely that this disparity is attributable simply to technical factors: the WM changes delineated were based on robust methodologies, for example the use of permutation testing which does not assume normality (Nichols and Holmes, 2002) and TFCE correction which is robust to nonstationarity (Smith and Nichols, 2009). WM changes more extensive than GM atrophy have been documented in previous WM tract studies of PPA syndromes, albeit inconsistently between studies (Acosta-Cabronero et al., 2011; Agosta et al., 2010, 2011; Galantucci et al., 2011; Schwindt et al., 2011); this inconsistency might be at least partly attributable to methodological differences and small patient cohorts. Moreover, in the present study, the apparent disparity in the distribution of GM and WM changes varied between PPA syndromes. One potential neurobiological explanation for these findings would hold that WM tract damage leads GM atrophy, at least in a proportion of PPA cases; and further, that this tract vulnerability might be more marked in certain PPA syndromes. A key pathophysiologic role of early WM alterations is suggested by previous neuroimaging evidence in asymptomatic mutation carriers (Borroni et al., 2008; Ringman et al., 2007) and histopathologic evidence of extensive WM pathology in frontotemporal lobar degeneration (FTLD) (Hiji et al., 2008; Neumann et al., 2007). To evaluate this issue fully would require a detailed longitudinal analysis of PPA syndromes, in order to establish the temporal evolution of regional GM atrophy in relation to WM tract disintegration.

Though the role of particular diffusivity metrics in defining neurodegenerative pathologies remains controversial we have replicated findings from a number of other studies (Acosta-Cabronero et al., 2010; Agosta et al., 2011); see Table 3). Together this evidence suggests that diffusivity metrics might vary in their potential to track WM disintegration in different PPA syndromes. For example in this study, across all groups, RD seemed to show the most widespread profile of WM change, and AX showed the least extensive change. These findings are in keeping with previous evidence in PPA (Galantucci et al., 2011; Schwindt et al., 2011). Tracts with the most marked involvement on voxel-wise analysis (e.g., UF and ILF in sv-PPA; see Table 2) tended to show broadly similar patterns of involvement across diffusivity metrics. This suggests that the sensitivity of metrics for detecting WM change might relate to the extent of tract damage, or that particular metrics (e.g., RD; see Table 2) might detect change earlier or might be intrinsically more sensitive to particular tissue pathologies (such as, potentially, AX for AD pathology, because AX revealed more extensive damage in the lv-PPA and AD disease control groups (see Supplementary Fig. 1) compared with nv-PPA or sv-PPA subtypes in this and previous studies (Galantucci et al., 2011; Josephs et al., 2006; Rohrer et al., 2011). We do not suggest that changes in any 1 diffusivity metric are intrinsically specific for a particular pathology, but rather, that some metrics or combinations of metrics might offer greater sensitivity in the detection of disease, which might in turn depend on how a particular pathological protein damages a tract (Acosta-Cabronero et al., 2010; Song et al., 2002). Again, these preliminary observations require substantiation in longitudinal studies with head-to-head comparisons of candidate diffusivity metrics and pathologic correlation.

The WM tract signatures here were identified at the level of large-scale networks; no single tract showed specificity for a particular syndrome (nor by inference, for a particular pathology). Comparing tract signatures across disease groups (Figs. 1, 2, and 3), sv-PPA was associated with predominant ventral tract involvement, nv-PPA with more anterior-dorsal tract involvement, and lv-PPA with more widespread tract changes. These WM tract profiles accord with a recent macroanatomical classification scheme proposed for pathologies in the FTLD spectrum, based on profiles of gray matter loss (Rohrer et al., 2011) and further suggest that disease specificity might lie in the pattern of large-scale network breakdown. This study corroborates previous work suggesting that the overall profile of tract involvement might signal particular pathologies. For example, the fornix and CB were here most markedly involved in lv-PPA; these structures have been identified as potentially useful anatomic biomarkers in the detection of AD pathology, which underpins a high proportion of lv-PPA cases (Huang et al., 2012; Oishi et al., 2012). Other tracts (for example, UF) were involved across syndromes; such tracts could potentially play a critical role in the diffusive spread of pathogenic proteins, which might constitute a common mechanism of network disintegration in neurodegenerative diseases (Raj et al., 2012; Warren et al., 2012; Zhou et al., 2012).

This study has several limitations and highlights directions for future work. Patient numbers were relatively small, assessments were exclusively cross-sectional, and histopathologic confirmation was not obtained. A more complete understanding of the WM tract correlates of the PPA syndromes will require larger disease cohorts followed longitudinally. This is particularly pertinent given the propensity of PPA syndromes to transform and converge over time (Rogalski et al., 2011b); this transformation might depend on tract alterations. A further important objective of future longitudinal studies (particularly targeting genetic cases) will be to relate earliest tract alterations to the onset and progression of neuronal loss and clinical disease; this in turn will require detailed behavioral correlation. To establish their specificity for proteinopathies and syndromes, in vivo tractographic profiles will ultimately need histopathologic correlation. From a clinical perspective, there is a need to establish the utility of particular diffusivity metrics as biomarkers and the validity and limitations of group-level patterns in classifying individual patients within each group (Dickerson et al., 2008). Larger patient cohorts might help offset overlap between PPA syndromes, however additional metrics derived from other modalities (e.g., functional MRI) might be needed to further refine distributed structural tractographic profiles and to identify tracts that are core to the development of each syndrome. Technical limitations of this work include the use of a study-specific WM skeleton under TBSS (restricting statistical analysis to the center of each tract), and the use of masks (which are somewhat arbitrary and might not capture the entire tract), particularly in areas of crossing fibers (Jbabdi et al., 2010). A further crucial issue which was not directly addressed here is the relation between tract degeneration and GM atrophy. Taking these caveats into account, the present findings support the concept of differentially vulnerable neuronal networks in particular PPA syndromes and proteinopathies. DTI metrics might furnish novel markers of brain network breakdown in these diseases; more fundamentally, the tractographic delineation of PPA syndromes might contribute new information about the pathophysiology of language network breakdown and underlying molecular architecture.

## Disclosure statement

The authors declare no actual or potential conflicts of interest.

Ethical approval for the study was obtained from the local institutional ethics committee and all subjects gave written informed consent to participate in accordance with the Declaration of Helsinki.

## Figures and Tables

**Table 1:** Summary of group demographic, neuropsychological, and CSF data

*n* (female)	nv-PPA		lv-PPA		sv-PPA		AD		Control	
	13 (10)		10 (5)		10 (7)		20 (11)		20 (12)	
	Mean	SD	Mean	SD	Mean	SD	Mean	SD	Mean	SD
Age	65.7	9.4	67.0	6.1	63.4	6.7	62.8	5.0	64.7	5.5
Disease duration (years)	3.3	1.2	4.0	1.4	5.0	2.0	5.6	3.8	—	—
Recognition Memory-Words (/50)^a^	40.5	8.9	29.9	9.1	32.0	8.0	30.1	6.1	47.1^b^	2.3
MMSE (/30)	18.0	10.9	16.9	8.5	20.6	8.5	21.0	4.6	29.6^b^	0.5
Recognition Memory-Faces (/50)^c^	36.7	5.8	29.8	7.4	31.7	7.8	33.7	6.0	41.8^b^	5.0
BPVS (/150)^d^	120.6	41.0	87.4	46.4	65.6	49.2	135.5	22.8	147.8^b^	1.5
Concrete synonyms (/25)^b^	18.3	4.6	17.8	5.6	14.3	6.3	—	—	24.5^b^	0.7
Graded Naming Test (/30)^e^	10.8	10.6	4.4	4.3	1.6	4.3	13.4	8.7	26.8^b^	1.7
Graded Arithmetic Test (/24)^f^	4.2	2.6	0.1	0.3	8.3	8.8	5.1	4.3	12.9^g^	4.4
VOSP Object Decision (/20)	16.5	3.1	14.6	6.2	14.6	4.3	15.7	2.5	18.0^b^	3.5
Stroop Color Naming (s)^h^	74.5	21.4	68.0	20.6	56.8	23.0	55.2	18.6	30.2^b^	3.8
Word Repetition (/45)^i^	21.8	16.0	40.3	5.1	43.9	1.4	—	—	44.3^g^	1.0
Sentence Repetition (/10)^j^	3.7	3.9	6.2	3.1	9.6	0.7	—	—	9.8^g^	0.6
Receptive Grammar-PALPA 55 (/24)	16.1	7.4	11.6	6.0	21.1	3.0	—	—	23.5^b^	0.7
Baxter Spelling Test (/30)	11.2	9.7	6.7	5.9	14.0	10.4	—	—	26.9^g^	1.3
CSF	*n* = 7		*n* = 9		*n* = 5		*n* = 10			
CSF Total tau (pg/mL)	540	370	888	318	350	131	770	355		
CSF ABeta_1–42_	384	148	256	122	669	156	240	64		

Number with neuropsychological data: sv-PPA, *n* = 9; nv-PPA, *n* = 11; lv-PPA, *n* = 10. Further background information about behavioral tests is provided in Supplementary data (see “Description of behavioural tests”). Key: AD, Alzheimer's disease; BPVS, British Picture Vocabulary Scale; CSF, cerebrospinal fluid; lv-PPA, logopenic variant PPA; MMSE, Mini-Metal State Examination; nv-PPA, non-fluent/agrammatic PPA; PALPA, psycholinguistic assessment of language processing in aphasia; PPA, primary progressive aphasia; sv-PPA, semantic variant PPA; VOSP, visual object and space perception test.

a *p* < 0.05 nv-PPA versus AD.

b *p* < 0.05 control versus all disease groups *p* < 0.05.

c *p* < 0.05 nv-PPA versus lv-PPA and sv-PPA; *p* < 0.05 lv-PPA versus sv-PPA.

d *p* < 0.05 sv-PPA versus all groups; *p* < 0.05 lv-PPA versus nv-PPA and AD.

e *p* < 0.05 sv-PPA versus all groups; *p* < 0.05 lv-PPA versus AD.

f *p* < 0.05 lv-PPA versus all groups.

g *p* < 0.05 control versus nv-PPA and lv-PPA.

h *p* < 0.05 nv-PPA versus AD.

i *p* < 0.05 nv-PPA versus lv-PPA and sv-PPA.

j *p*< 0.05 sv-PPA versus nv-PPA and lv-PPA.

**Table 2:** White matter tract changes in PPA groups compared with healthy control group: quantitative data

Group	Fractional anisotropy				Axial diffusivity				Radial diffusivity				Trace diffusivity			
	Tract	*p*	Voxels	%	Tract	*p*	Voxels	%	Tract	*p*	Voxels	%	Tract	*p*	Voxels	%
sv-PPA	R UF	**0.002**	309	17.9	L UF	**0.004**	405	14.5	R UF	**0.001**	371	21.4	R UF	**0.003**	364	21.0
	L UF	**0.003**	526	18.9	L ILF	**0.004**	1113	11.7	L UF	**0.002**	566	20.3	L UF	**0.004**	573	20.5
	R CB	**0.004**	243	13.9	L ATR	0.01	308	2.7	R CB	**0.006**	205	11.7	L ILF	**0.008**	1508	15.9
	L ILF	**0.008**	1265	13.3	R ILF	0.01	685	10.3	L ILF	**0.006**	1621	17.1	R ILF	0.010	1025	15.3
	L ATR	0.008	133	1.2	R UF	0.02	257	14.8	L SLF	**0.006**	1286	9.3	L SLF	0.012	1200	8.7
	CC	0.01	8694	9.6	CC	0.02	3268	3.6	R ILF	**0.008**	1077	16.1	L ATR	0.013	537	4.7
	R ILF	0.01	767	11.5	R SLF	0.02	67	0.6	L ATR	0.01	584	5.1	R ATR	0.017	312	3.0
	L CB	0.01	244	8.0	L SLF	0.02	542	3.9	CC	0.01	8139	9.0	CC	0.020	7578	8.3
	L SLF	0.01	1229	8.9	R ATR	0.02	108	1.1	R ATR	0.01	378	3.7	Fornix	0.022	349	3.3
	R SLF	0.02	853	7.7	L CST	0.03	196	2.7	L CST	0.01	221	3.0	R SLF	0.029	608	5.5
	L CST	0.02	17	0.2	R CST	0.04	147	2.2	Fornix	0.01	330	3.1	R CB	0.031	83	4.7
	Fornix	0.02	45	0.4	Fornix	0.04	12	0.1	R SLF	0.01	831	7.5	L CST	0.032	206	2.8
	R ATR	0.03	36	0.4	—	—	—	—	L CB	0.02	338	11.1	L CB	0.037	202	6.6
nv-PPA	L UF	**0.005**	334	12.0	L ATR	**0.007**	672	5.865	L ATR	**0.007**	672	5.87	L ATR	**0.009**	644	5.6
	L CST	**0.009**	47	0.6	L CST	0.01	312	4.251	L UF	**0.008**	395	14.2	L UF	0.011	323	11.6
	CC	0.01	7334	8.1	L UF	0.02	71	2.545	CC	0.01	7853	8.65	CC	0.011	7184	7.9
	L CB	0.01	3	0.1	CC	0.02	4164	4.585	R ATR	0.01	406	3.96	R ATR	0.012	300	2.9
	R CST	0.01	19	0.3	L SLF	0.02	15	0.109	L CST	0.01	49	0.67	R CST	0.016	106	1.6
	Fornix	0.01	33	0.3	R CST	0.02	267	3.979	R UF	0.01	134	7.74	R UF	0.017	111	6.4
	L ATR	0.01	618	5.4	R UF	0.03	66	3.813	R SLF	0.01	16	0.14	Fornix	0.021	20	0.2
	R ATR	0.01	288	2.8	R ATR	0.03	111	1.083	Fornix	0.02	33	0.31	L CB	0.024	18	0.6
	R UF	0.02	90	5.2	Fornix	0.04	10	0.095	R CST	0.02	16	0.24	R CB	0.025	1	0.1
	R SLF	0.02	17	0.2	L CB	0.04	7	0.23	L SLF	0.02	752	5.44	R SLF	0.032	28	0.3
	R ILF	0.02	15	0.2	R SLF	0.05	210	0.135	R CB	0.02	99	1.20	L CST	0.033	40	0.5
	L ILF	0.03	78	0.8	—	—	—	—	L CB	0.03	21	0.53	L SLF	0.034	77	0.6
	L SLF	0.03	595	4.3	—	—	—	—	L ILF	**0.04**	98	1.03	—	—	—	—
	R CB	0.04	15	0.9	—	—	—	—	R ILF	**0.04**	27	0.40	—	—	—	—
lv-PPA	Fornix	**0.006**	41	0.388	L ATR	**0.004**	724	6.319	L ILF	**0.003**	1888	19.89	L CB	**0.002**	230	7.6
	L ILF	**0.009**	1628	17.15	L SLF	**0.004**	1303	9.432	R ILF	**0.004**	1403	21	L ILF	**0.002**	1848	19.5
	L CB	**0.009**	392	12.9	L ILF	**0.004**	1381	14.55	CC	**0.004**	11278	12.42	CC	**0.004**	12320	13.6
	L UF	**0.009**	459	16.45	CC	**0.005**	9711	10.69	L UF	**0.005**	394	14.12	R ILF	**0.004**	1349	20.2
	CC	0.010	9711	10.69	L CB	**0.005**	172	5.66	L CB	**0.005**	374	12.31	R UF	**0.005**	241	13.9
	L ATR	0.012	526	4.591	L CST	**0.006**	276	3.76	Fornix	**0.005**	45	0.426	L SLF	**0.005**	1577	11.4
	L SLF	0.012	788	5.704	R ILF	**0.006**	994	14.88	L SLF	**0.006**	1470	10.64	L UF	**0.005**	366	13.1
	R CB	0.013	245	14	R SLF	**0.006**	913	8.227	R ATR	**0.006**	351	3.423	L ATR	**0.005**	728	6.4
	R UF	0.014	145	8.377	R CST	**0.007**	190	2.831	R UF	**0.006**	237	13.69	Fornix	**0.007**	50	0.5
	R ILF	0.015	1085	16.24	R UF	**0.007**	233	13.46	L ATR	**0.007**	711	6.206	R SLF	**0.008**	1012	9.1
	L CST	0.019	223	3.038	L UF	**0.009**	262	9.391	R CB	**0.008**	237	13.54	R ATR	**0.008**	364	3.6
	R ATR	0.019	300	2.926	R ATR	0.011	281	2.741	R SLF	0.010	979	8.821	R CB	**0.009**	41	2.3
	R CST	0.020	284	4.232	Fornix	0.019	9	0.085	L CST	0.013	253	3.447	R CST	0.010	209	3.1
	R SLF	0.032	719	6.479	R CB	0.022	17	0.971	R CST	0.015	233	3.472	L CST	0.011	192	2.6

% indicates percentage of affected voxels within each tract. All results are FWE corrected *p* < 0.05 and ordered by *p* value. Bold indicates a significance level of *p* < 0.01. Key: ATR, anterior thalamic radiation; CB, cingulum bundle; CC, corpus callosum; CST, corticospinal tract; ILF, inferior longitudinal fasciculus; L, left; lv-PPA, logopenic variant primary progressive aphasia; nv-PPA, nonfluent/agrammatic primary progressive aphasia; PPA, primary progressive aphasia; R, right; SLF, superior longitudinal fasciculus; sv-PPA, semantic variant primary progressive aphasia; UF, uncinate fasciculus.

**Table 3:** Comparison of fractional anisotropy changes in DTI studies of PPA

Report	Subjects	Tract selection	Dir	Statistical method	Software	UF			ILF			SLF			Other		
						NV	LV	SV	NV	LV	SV	NV	LV	SV	NV	LV	SV
Agosta et al., 2010	SV = 5; control = 8	ROI (L ILF/L SLF/L UF/CC)	55	Linear mixed effects model	In-house	nt	nt	L	nt	nt	nsr	nt	nt	L^a^	nt	nt	CC^b^
Whitwell et al., 2010	NV = 7; SV = 4; control = 19	ROI; *n* = 16	21	ANCOVA	DTI Studio	nsr	nt	nsr	nsr	nt	L^c^	R^d^	nt	nsr	nt	nt	nt
Galantucci et al., 2011	NV = 9; LV = 9; SV = 9; control = 21	ROI (ILF/UF/SLF)	64	ANCOVA	PROBTRACKX	nsr	nsr	B	nsr	nsr	B^e^	L	L^f^	L^g^	nt	nt	nt
Acosta-Cabronero et al., 2011	SV = 10; control = 21	Whole brain	63	Nonparametric permutation test	FDT/TBSS/Tractography	nt	nt	nsr	nt	nt	L^h^	nt	nt	nsr	nt	nt	nt
Agosta et al., 2011	NV = 9; LV = 4; SV = 7; control = 27	Whole brain	32	Nonparametric permutation test	FDT/TBSS	L	nsr	B	L^i^	nsr	L^i^	L	nsr	nsr	B CB, CC, FO	FO	CC, L CB, FO
Schwindt et al., 2011	SV = 9; NV = 9; control = 16	Whole brain	23	Nonparametric permutation test	FDT/TBSS	L	nt	B	L	nt	B	nsr	nt	B	nt	nt	L ATR, B FM, CC
Present study	NV = 13; LV = 10; SV = 10; AD = 20; control = 20	Whole brain	64	Nonparametric permutation test	CAMINO/TBSS	L	L	B	nsr	L	L	nsr	nsr	nsr	L CST	L CB, FO	L ATR, R CB

Results reported reflect most statistically robust findings (for present study, only significance values *p* < 0.01 are included here; see also Table 2). Key: AD, Alzheimer's disease; ANCOVA, analysis of covariance; ATR, anterior thalamic radiation; B, bilateral; CB, cingulum bundle; CC, corpus callosum; CST, corticospinal tract; Dir, number of diffusion gradient directions used in DTI acquisition; DTI, diffusion tensor imaging; FDT, FMRIB diffusion toolbox; FM, forceps major; FO, fornix; ILF, inferior longitudinal fasciculus; L, left; LV, logopenic variant PPA; nsr, no significant result found; nt, data not reported/tested; NV, nonfluent/agrammatic PPA; PPA, primary progressive aphasia; R, right; ROI, region of interest; SLF, superior longitudinal fasciculus; SV, semantic variant; TBSS, tract based spatial statistics; UF, uncinate fasciculus.

a Arcuate and frontoparietal SLF.

b Genu.

c Posterior ILF.

d Anterior SLF.

e Bilateral anterior ILF and left medial ILF.

f SLF temporoparietal fibers.

g Arcuate and SLF temporoparietal.

h Results reported based on authors' figures.

i Inferior fronto-occipital fasciculus.

## Appendix A Supplementary data

>Supplementary Table 1
