# Supplementary material for: White matter tract signatures of the progressive aphasias
Source: Neurobiol Aging. 2013 Jun;34(6):1687–99. doi: 10.1016/j.neurobiolaging.2012.12.002 (PMC3601331; doi:10.1016/j.neurobiolaging.2012.12.002)
Supplement: >Supplementary Table 1 [file mmc1.pdf]

Supplementary Table 1.

White matter tract changes in PPA groups compared to AD group: quantitative data

White Matter changes in sv-PPA vs. AD											
Axial Diffusivity (sv > AD)				Radial Diffusivity (sv > AD)				Trace Diffusivity (sv > AD)			
Tract	p-value	voxels	%	Tract	p-value	voxels	%	Tract	p-value	voxels	%
**L UF**	**0.003**	**132**	**4.73**	**L ILF**	**0.01**	**512**	**5.39**	**L ILF**	**0.01**	**463**	**4.88**
**L ILF**	**0.007**	**393**	**4.14**	L UF	0.012	385	13.80	L UF	0.02	298	10.68
R UF	0.011	90	5.20	R ILF	0.015	236	3.53	R ILF	0.02	214	3.2
CC	0.015	18	0.02	R UF	0.019	207	11.96	R UF	0.02	162	9.36
R ILF	0.018	213	3.19	CC	0.035	12	0.01	CC	0.02	7	0.01

White Matter changes in AD vs. nv-PPA			
Axial Diffusivity (AD > nv)			
Tract	p-value	voxels	%
CC	0.039	1244	1.37
Fornix	0.039	2	0.02
R ILF	0.045	216	3.23
R SLF	0.047	36	0.32
