# Supplementary material for: White matter tract signatures of the progressive aphasias
Source: Neurobiol Aging. 2013 Jun;34(6):1687–99. doi: 10.1016/j.neurobiolaging.2012.12.002 (PMC3601331; doi:10.1016/j.neurobiolaging.2012.12.002)
Supplement: Supplementary Table 2 [file mmc2.pdf]

Supplementary Table 2. White matter tract comparisons between PPA groups: quantitative data

	sv-PPA > nv-PPA				sv-PPA > lv-PPA				lv-PPA > sv-PPA				lv-PPA > nv-PPA			
	Tract	p-	voxels	%	Tract	p-	voxels	%	Tract	p-	voxels	%	Tract	p-	voxels	%
Axial Diffusivity	**L UF**	**0.004**	**129**	**4.6**	**L UF**	**0.009**	**125**	**4.5**	CC	0.03	1496	1.6	R ILF	0.01	998	14.9
	L ILF	0.012	622	6.6	L ILF	0.01	190	2.0	L CST	0.05	25	0.3	L ILF	0.01	1565	16.5
	R ILF	0.023	303	4.5									CC	0.02	4427	4.9
	R UF	0.024	67	3.9									L SLF	0.02	817	5.9
	CC	0.025	102	0.1									R CST	0.02	437	6.5
													R ATR	0.02	63	0.6
													R SLF	0.02	848	7.6
													L CB	0.02	66	2.2
													L ATR	0.02	31	0.3
													L CST	0.02	271	3.7
													R UF	0.04	42	2.4
Radial Diffusivity	L UF	0.011	314	11.3	L UF	0.02	271	9.7					L ILF	0.02	1262	13.3
	L ILF	0.015	516	5.4	L ILF	0.02	278	2.9					L SLF	0.03	381	2.8
	R ILF	0.019	309	4.6									CC	0.03	2601	2.9
	R UF	0.020	109	6.3									R CST	0.04	245	3.7
	CC	0.030	6	0.0									R ILF	0.04	794	11.9
													R ATR	0.04	30	0.3
													R SLF	0.05	93	0.8
Trace Diffusivity	L UF	232	8.32	0.01	L UF	0.01	144	5.16					L ILF	0.01	1636	17.23
	L ILF	556	5.86	0.02	L ILF	0.02	259	2.73					CC	0.01	4452	4.90
	R ILF	280	4.19	0.02									R ILF	0.01	1150	17.21
	R UF	88	5.08	0.02									L CB	0.01	170	5.59
	CC	13	0.01	0.02									L SLF	0.01	813	5.89
													R SLF	0.02	372	3.35
													R UF	0.02	29	1.68
													Fornix	0.02	5	0.05
													R CST	0.03	490	7.30
													L UF	0.03	30	1.08
													L CST	0.03	262	3.57
													L ATR	0.04	45	0.39
													R ATR	0.04	126	1.23
FA													L ILF	0.04	85	0.9
													CC	0.05	170	0.2
													R CST	0.05	68	1.0
